# Professor Doctor Carol Davila, General: the founder

**Published:** 2009

**Authors:** Florian Popa, Dan Mischeanu, Victor Lorin Purcarea

**Affiliations:** * „Carol Davila” University of Medicine and Pharmacy, Bucharest, Romania; ** Urology Clinic, “Dr. Carol Davila” Clinical Central Military Emergency Hospital, Bucharest, Romania

On August 24, 2009 there will have been 125 years from the death of Professor Doctor Carol Davila, General.

This article could have had the name “The Merits of Carol Davila” or “The Heritage of Carol Davila” or “A MAN for a nation”. It is clear that Professor Doctor Carol Davila, General was the founder of many institutions, contributing significantly and decisively to the progress of the Romanian society, permanently living and thinking to the benefit of the patients. He was an astonishing personality who gained the patients’ over and remained in their minds for always, due to the realization of the equality between promise and deed.

He was an exceptional doctor, founder of the National School of Medicine and Pharmacy, who dedicated himself to his job bringing rigueur and professionalism where only good intentions and amateurism were present, creating an ordered, meaningful structure, in which he was able to realize whatever his visionary spirit had thought and wanted, thanks to his exceptional will and tenacity.

The man whose visiting card, at the end of his life, was composed of only one word: DAVILA, was born on April 8, 1828 in Avila, Italy, near Parma. His name was believed to be Charles Antoine Francois by some; others believed that his name was Carlos Antonio Francesco d’Avila. Some sources state the fact that he was the son of the musician Franz Liszt and the Countess d’Agoult and others say he was the son of the same Liszt (who was only 17 years old in 1828?!) and Mrs. Crig (or de Krick). However, it is certain that Carol Davila did not enjoy talking about “biographies”.

He lived in Frankfurt-Main, Germany his first 10 years of life, and then he went to Limoges, France (where he began studying French in high school). Between 1843 and 1845 he ended up in Nantes, as a student trainee, in Leon le Sant’s drugstore.

It is probable that this was the moment he started to think with pleasure to the pharmaceutical techniques and to which, later, have become the famous “Davila drops” – the tincture made up of peppermint oil, rhubarb, alcoholic extract of cinnamon and tincture of opium, which were beneficial in reducing vomiting in cholera.

After 1845 he continued the high school in Angers, graduating in 1847. He worked as a stagier pharmacist in C. S. Ollivier’s drugstore. He took medical instructions and graduated from high school in the so-called “Preparatory School of Medicine and Pharmacy” (November 1, 1847-1850). In order for him to become a Medical Doctor he had to continue the studies at the Faculty of Medicine, in Paris.

In 1853, he was graduating that university presenting his PhD diploma "From Prophylaxis to Syphilis”, in spite of some financial difficulties.

Sometimes, it is said that “the end of an adventure is only the beginning of another one and that everything has an explanation in the end!”

The Prince of Wallachia, Barbu Stirbei made an announcement in which he was asking for a doctor to organize the medical service of the army and, in the same time to be a personal doctor of the ruler. The French consul Bechard (married to one of Lascar Catargiu’s daughters, and son of the Dean of the Faculty of Medicine in Paris), made this announcement public in the capital of France, and the person recommended by the Dean for the job was Carol Davila himself. He reached Wallachia on March 13, 1853 and enters it from Giurgiu. He would remain on that territory for always (it is said that he had had a request to become a doctor at the Persian Chess’ Manor!, which he declined). He amazed the state leadership from the very beginning- he disappeared without any notice for 3 days leaving in his first sanitary inspection and came back with a strong project of sanitary reform. At first, he was given shelter in a humble place, full of damp and very dark on the dock of Dambovita. Due to that place he suffered from joint rheumatism, he ended up with stiffen of the right elbow. This explains his habit of keeping his right arm at the back- a thing which was also surprised by the sculptor Carol Storck while making the famous statue of Carol Davila in front of the Faculty of Medicine and Pharmacy (this statue was inaugurated on October 13, 1903).

**Fig. 1:**
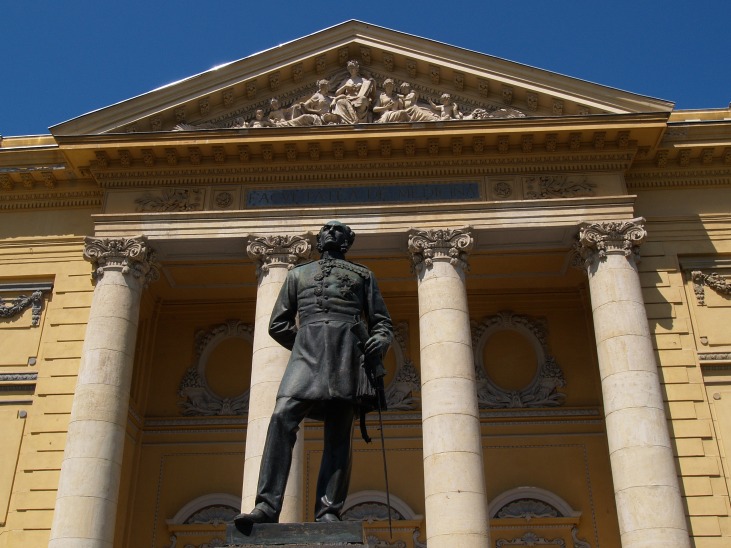
The statue of Carol Davila in front of the Faculty of Medicine and Pharmacy, in Bucharest (made by Carol Storck in 1903)

The fact that Carol Davila came to Wallachia was an adventure, a chance or destiny? No matter what it had been, his accomplishments remain over the years and represent great icons of a nation which was at the beginning of the road.

The doctor Carol Davila conceived a functional medical system by organizing both the military and the civil services. 

Soon after his arrival in Wallachia he was made Chief at the Hospital of the Army of Mihai Voda where he introduced “the spirit of discipline and the sense of responsibility”, new ways of treatment (blood transfusion – for the first time in our country) and anti smallpox vaccination. In 1856 he was made supra-doctor of the army and his hiring contract was prolonged for another 6 years.

In the summer of 1859 Dr. Carol Davila, General (made General by Al. I. Cuza) was made Superior Doctor of all the troupes in the principality, by a decree published in “Monitorul Oficial” and, in 1862 he was named the Chief of the Sanitary Service of the Unified Country (equivalent to the function of Ministry of Health).

**Fig. 2:**
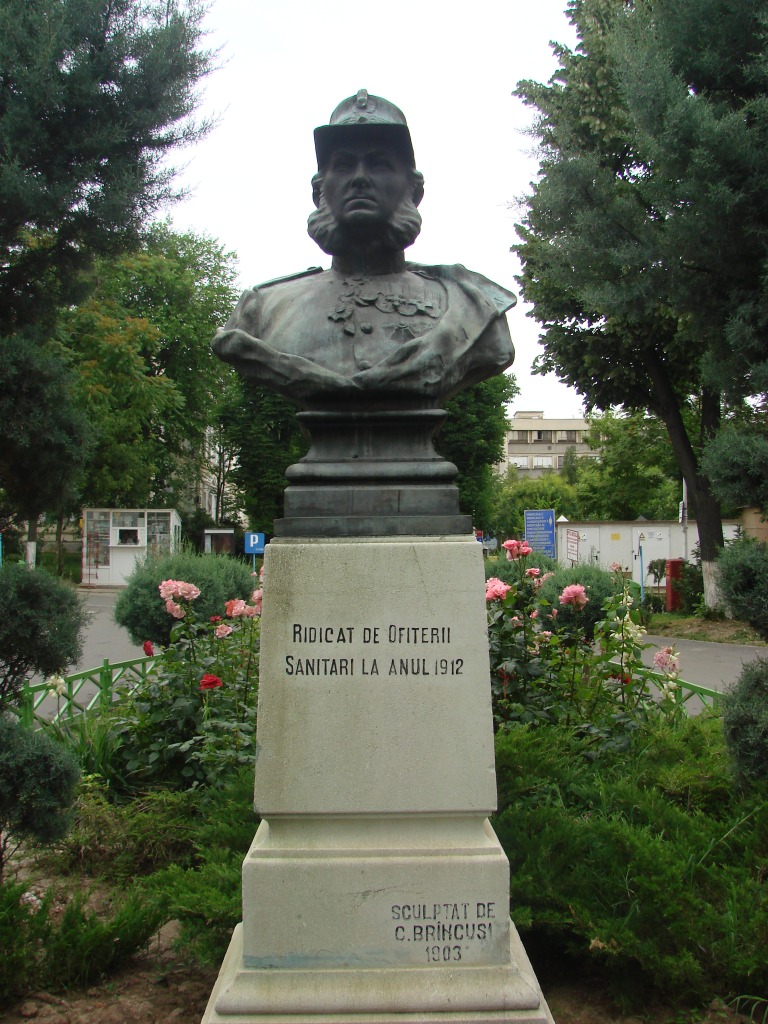
The bust of Carol Davila in the courtyard of the Central Clinical Military Emergency Hospital (made by Constantin Brancusi)

The following words of Carol Davila must be learnt: “Finding the new country, Romania, on the shores of the Danube, the France of the Orient, as far as traditional ideas and civilization aspirations of the bigger sister are concerned, I have become both with my heart and my action, a Romanian citizen”.

However, Carol Davila’s bigger accomplishment is the reorganization and the edification of the Romanian medical education. He proposed and turned the Little School of Surgery into the National School of Medicine and Pharmacy (in 1857, a fact which was highlighted by the royal decree 1092/ 1857) and, in 1869 it became the Faculty of Medicine and Pharmacy in Bucharest. The diplomas given in Bucharest were and still are recognized in France and Italy. 

**Fig. 3:**
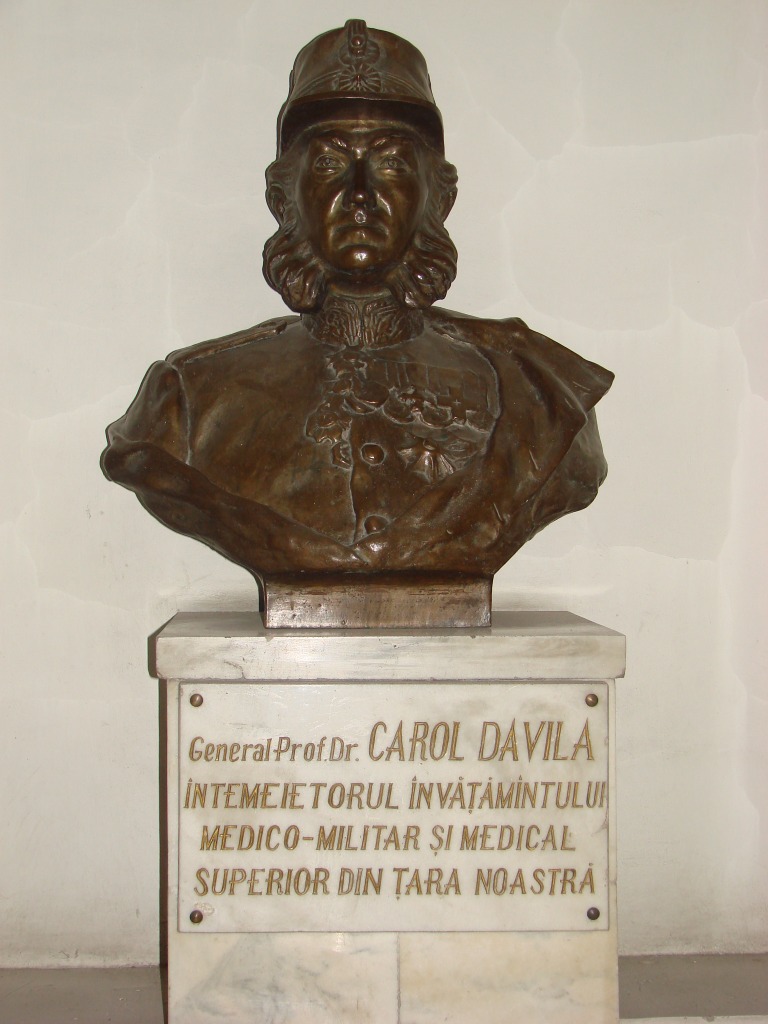
The bust of Carol Davila in the Medical Direction area, Bucharest

He had preoccupations of internal medicine, epidemiology, orthopedics and abdominal surgery; he introduced modern treatments in the hospitals as well as the laboratory tests. 

He created at the Central Military Hospital the first laboratory of chemical analyses. He contributed to the apparition of the first medical journal “Romanian Pharmacopoeia” and he founded the Red Cross Society in Romania. He introduced, just like in France, the exams for admission and graduation from the university and the obligatoriness of the doctorate papers. He contributed to the founding of the Botanic Garden in Bucharest having a triple purpose: knowledge, relaxation and rest but also the plant resource for pharmaceutical substances!. He founded “Elena Doamna” asylum and lots of orphanages. Moreover, he was the health adviser for three important leaders: Barbu Stirbei, Alexandru Ioan Cuza and Carol I. Many of his students have become, over the years, teachers of the Faculty of Medicine and Pharmacy in Bucharest. 

He also had Masonic preoccupations. Taking into account some sources, he was initiated in 1874 and shortly after, he became Venerable Master of the Freemasons’ Lodge in Heliopolis. In 1875 he became President of the Masonic Committee for the helping of the citizens of Bucharest affected by the floods. 

Between 1879 and 1884 he was Pro Great Master of the Big Orient of Romania. 

He had an uncurbed character, sometimes with choleric accents; he was a good organizer with a prodigious intelligence. 

He had not been kept away from trouble. His second wife Ana Davila (born Racovita) died because of a medical malpractice - taking strychnine instead of quinine, prescribed by a colleague of the General. Until his death (due to cardiac causes), left alone with his 4 children (Alexandru, Zoe, Elena and Pia), he continued to work with self-sacrifice and devotion. 

His resting place is in a tomb on the hill of Cotroceni (in fact the hill of Tacalia).

**Fig. 4:**
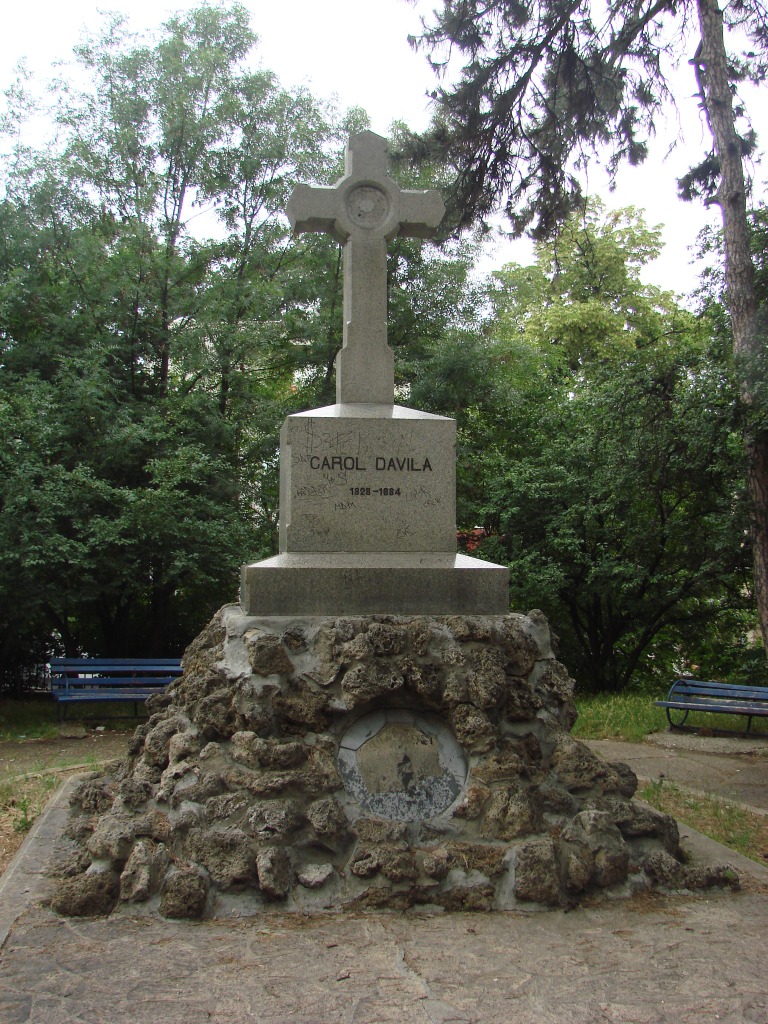
The tombstone of Carol Davila on the Hill of Tacalia

Analyzing the data and looking back at the accomplishments of Dr. Carol Davila, General, we can conclude, referring to the character of the man that: he was tenacious, calm and patient in realizing his objectives, he appreciated the truth and precision, he often made proof of modesty and was loyal to the authority of the time. 

Today, the University of Medicine and Pharmacy in Bucharest, Carol Davila University Foundation and Printing House, the Central Clinical Military Emergency Hospital and the Nephrology Clinical Hospital in Bucharest all bear his name, with great devoutness and gratitude. 

**Fig. 5:**
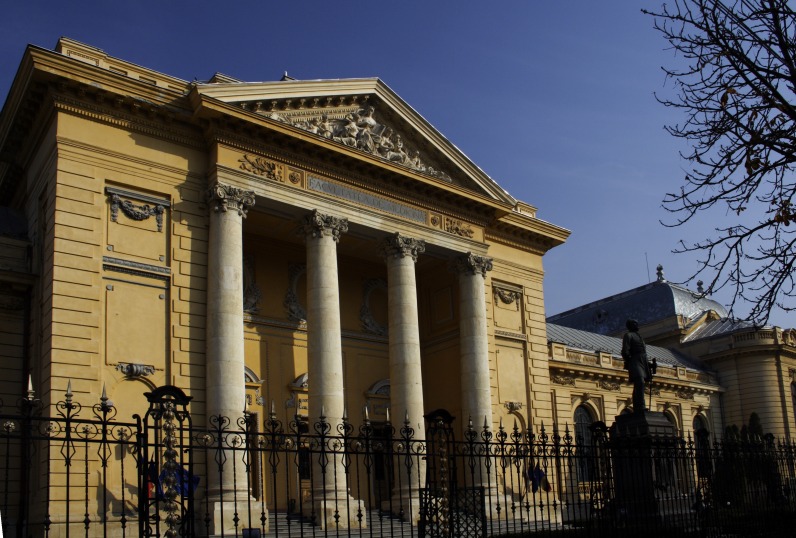
The Building of “Carol Davila” University of Medicine and Pharmacy in Bucharest

What should not be omitted the moment we bring to discussion the ACTS of Carol Davila is his intelligence, his providential organizational spirit, his culture which was inspired from many sources, his prudence and the flexibility of his spirit perfectly adapted to the character of the Romanian people, in which he had fully integrated and, last but not least, his diligence and courage to implement and carry out so many projects.
